# The application of artificial intelligence in glaucoma diagnosis and prediction

**DOI:** 10.3389/fcell.2023.1173094

**Published:** 2023-05-04

**Authors:** Linyu Zhang, Li Tang, Min Xia, Guofan Cao

**Affiliations:** ^1^ The Affiliated Eye Hospital of Nanjing Medical University, Nanjing, China; ^2^ The Fourth School of Clinical Medicine, Nanjing Medical University, Nanjing, China

**Keywords:** Glaucoma, artificial Intelligence, visual field, optical coherence tomography, fundus photographs

## Abstract

Artificial intelligence is a multidisciplinary and collaborative science, the ability of deep learning for image feature extraction and processing gives it a unique advantage in dealing with problems in ophthalmology. The deep learning system can assist ophthalmologists in diagnosing characteristic fundus lesions in glaucoma, such as retinal nerve fiber layer defects, optic nerve head damage, optic disc hemorrhage, etc. Early detection of these lesions can help delay structural damage, protect visual function, and reduce visual field damage. The development of deep learning led to the emergence of deep convolutional neural networks, which are pushing the integration of artificial intelligence with testing devices such as visual field meters, fundus imaging and optical coherence tomography to drive more rapid advances in clinical glaucoma diagnosis and prediction techniques. This article details advances in artificial intelligence combined with visual field, fundus photography, and optical coherence tomography in the field of glaucoma diagnosis and prediction, some of which are familiar and some not widely known. Then it further explores the challenges at this stage and the prospects for future clinical applications. In the future, the deep cooperation between artificial intelligence and medical technology will make the datasets and clinical application rules more standardized, and glaucoma diagnosis and prediction tools will be simplified in a single direction, which will benefit multiple ethnic groups.

## 1 Introduction

Glaucoma alludes to a chronic neurodegenerative disease that is associated with progressive loss of optic disc edge, retinal nerve fiber layer (RNFL) thinning and ganglion cell damage. It has been the second most common cause of blindness globally (Davis et al., 2016; GBD, 2019 Blindness and Vision Impairment Collaborators, 2021). The number of people with glaucoma (aged 40-80 years) worldwide was estimated to be 76.0 million in 2020 and 111.8 million in 2040 (Tham et al., 2014). Because of the progressive and insidious nature of glaucoma, early diagnosis is extremely vital to prevent disease progression and permanent vision loss. However, identifying glaucoma is a complicated process that requires multiple examinations and clinical expertise, which would be time-consuming and labor-intensive. In resource-constrained and geopolitically disadvantaged places, this process is beset by several challenges, such as a lack of healthcare infrastructure, inadequate follow-up, and poor therapy adherence (Nduaguba and Lee, 2006; Santos Martins et al., 2021). Considering that many potential glaucoma patients are at risk of future vision persecution or even blindness, early identification of glaucoma and improvement of diagnostic practices are topics that modern ophthalmologists are constantly striving for. The emergence of artificial intelligence (AI) technology and its integration with ophthalmology has solved this problem to some extent.

**FIGURE 1:**
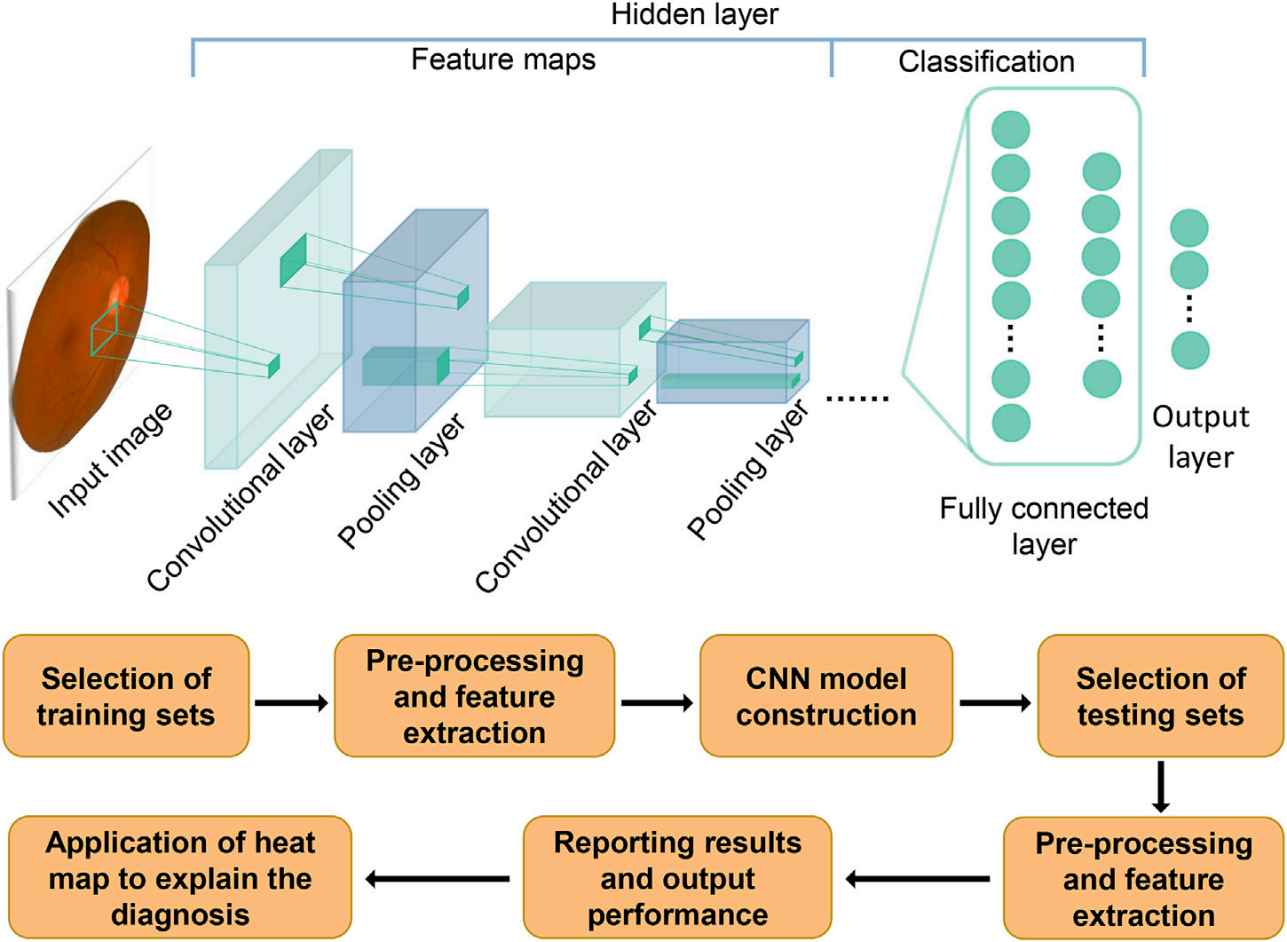
Schematic diagram of how the CNN model is generated and how it works. The flowchart below shows the process of constructing and evaluating a deep learning model with CNN structure. The schematic above depicts the process of how a CNN model works. After inputting an image, convolution and pooling are performed to extract the image features. Fully connected layers are used to classify the features, with all computations converging to the final model prediction in the output layer.

AI is expected to equip ophthalmologists with revolutionary automated methods for diagnosing and managing ocular illnesses. It is a multidisciplinary science that allows machines to simulate human cognitive processes such as learning, reasoning, problem solving, information processing, social awareness, and general intelligence (Hamet and Tremblay, 2017). It has the powerful data processing capability to analyze data and predict development trends autonomously. For autonomous diagnosis of characteristic fundus lesions of glaucoma, such as retinal nerve fiber layer defects, optic nerve head injury, and optic disc hemorrhage, it has unique advantages. Artificial intelligence encompasses machine learning, which learns and improves itself automatically from data, without the need for human-written programs to specify rules and logic. Machine learning in turn includes deep learning (DL). Deep learning, a development of machine learning within the field of AI, analyzes data using layered algorithmic frameworks. The architecture was inspired by the biological neural networks of animal brains (LeCun et al., 2015; Thompson et al., 2020). DL has relatively mature applications in bioengineering and smart medicine, and there are already successful cases in ophthalmology. In 2018 the U.S. Food and Drug Administration approved the first fully autonomous AI algorithm IDx-DR for the detection of diabetic retinopathy (Abràmoff et al., 2018), which has greatly advanced the development and dissemination of DL in the clinical setting. Recently, DL is evolving day by day, new network structures convolutional neural networks (CNN) have already made prominent contributions in a number of disciplines including dermatology (Combalia et al., 2022), cardiovascular disease risk prediction (Padmanabhan et al., 2019), radiology and ophthalmology (Setio et al., 2016; Xu et al., 2020b; Benet and Pellicer-Valero, 2022). Promising to provide ophthalmologists with novel tools for the diagnosis and treatment of ocular diseases. Figure 1 depicts a schematic diagram of a deep learning model with CNN structure to process images. With these technical conditions, ophthalmologists can greatly reduce the cumbersome process during diagnosis and treatment, decrease manpower resources for interpreting auxiliary examinations, and increase the ability to capture subtle lesions that are not discernible to human eyes.

Enhanced computing power expanded storage capacity, and compilation of medical data allow for broader applications and more accurate diagnostic methods for AI technology in the direction of disease screening and ancillary test judgment. In ophthalmology, due to the reliance on ancillary examination images and the requirement for various diagnostic evidence, AI is uniquely positioned to analyze and interpret glaucoma intraocular pressure, visual field (VF), optical coherence tomography (OCT) and retinal fundus images (Devalla et al., 2020). Detection systems using AI can overcome the stress of the healthcare resource shortage due to an aging population (Chan et al., 2016), provide mass screening at low cost, especially for regions lacking medical care professionals, and can provide full-cycle health monitoring for patients in a high-quality and efficient manner (Balyen and Peto, 2019). The current functional and structural tests commonly used in glaucoma diagnosis include VF detection, retinal fundus photography and OCT, so in this review article we detail the combination of these tests with AI for glaucoma diagnosis and prediction, as well as some of the other techniques under development for their current applications in research and clinical practice. We further explore the limitations and potential challenges, as well as the prospects for future applications in the hope of providing new perspectives for scholars in this field to contemplate.

## 2 Diagnostic model of glaucoma

### 2.1 Visual fields

In the clinical setting, VFs are widely used as the gold standard for diagnosing whether a patient is glaucomatous, and the Standard Automated Perimetry (SAP) remains the primary tool for diagnosing and tracking functional changes in the disease. SAP assessment of functional impairment rates is extremely important for establishing patient prognosis and treatment aggressiveness. However, this test is often influenced by a variety of subjective factors, such as patient attention fatigue or poor doctor-patient cooperation, which can readily influence the ultimate judgment of disease progression. AI systems combined with some advanced testing devices for review, such as the frequency-doubling perimetry, Humphrey Matrix 24-2 test, short-wavelength automated perimetry, and Heidelberg edge perimetry, can yield accuracy gains at lower cost and higher efficiency. Deep learning models use VFs collected from various healthcare facilities, commonly use total deviation plots, mean deviation values, and pattern deviation probability plots. Data samples with excessive false-positive and false-negative rates were excluded when collecting this VF information. Additional features of glaucoma patients were also collected as an assist and the plots were processed to make lesion features more susceptible to detection. The relevant information is extracted and used as variables in the classifiers to train algorithms for diagnosing or predicting glaucoma conditions. A few models also incorporate numerical pattern deviation plots and numerical displays to assist in training. Results obtained can be compared with clinicians’ judgments to verify their validity.

In 2014 (Asaoka et al., 2014) developed a Random Forests machine-learning method in order to distinguish the VFs of preperimetric open angle glaucoma eyes. After achieving good results in the area under the receiver operating characteristic curve (ROC) and significant total deviation differences with this method, studies combining AI with VF detection to diagnose glaucoma are beginning to gain traction. After that, other researchers developed a CNN system using the vision geometrical group (VGG) network structure (Li et al., 2018a). The VGG network is first pre-trained on the ImageNet dataset, then the output dimension of the penultimate layer is modified with the last layer output two-dimensional (2D) vector, and all parameters of the network are initialized and updated. The network is compared with the results of rule-based methods (like Advanced Glaucoma Intervention Study criteria and Glaucoma Staging System criteria) and non-deep machine learning algorithms (like random forest). With an accuracy of 0.876, specificity of 0.826, and sensitivity of 0.932, the CNN far exceeded several other types of AI visual field algorithms, showing good glaucomatous VF discrimination. The results demonstrate the advantages of the CNN algorithm for applications. However, one thing to note is that this study only used pattern deviation images as input objects and early glaucoma may not be identified, and the capabilities of DL models need to be expanded to diagnose more types of glaucoma.

Convolutional long short-term memory neural networks for glaucoma progression detection have also been trained and combined with clinical data (Dixit et al., 2021). This neural network extracts spatiotemporal features of glaucoma progression, and the researchers used a longitudinal dataset containing VF as well as clinical data to improve the evaluation of the model. The researchers utilized two machine learning models that defined progression using algorithms such as VF index slope, pointwise linear regression, and mean deviation slope. And one was trained using clinical data containing information such as cup-to-disc ratio, intraocular pressure and central corneal thickness. The long short-term memory neural network had an accuracy of 91%–93%, and the model trained using clinical data showed a higher AUC than the model trained using only the VF dataset. Creating clinically accessible diagnostic interfaces and windows is also essential. Huang et al. (Huang et al., 2022) developed a fine-grained grading deep learning system and an interactive diagnostic aid interface has been created for clinical implementation. Two DL models Humphrey Field Analyzer (HFA) data (FGG-H) and Octopus data (FGG-O) were constructed both using the Residual Neural Network (ResNet) structure, where the FGG-O was initialized with the final parameters of FGG-H preprocessed with HFA data from the Harvard dataset, and they added identity mapping over CNN to grade glaucoma with high accuracy. The fine-grained grading deep learning system achieves almost the same accuracy as ophthalmology clinicians and provides a user-friendly interface for patients and physicians to perform the test. In addition, a smartphone application-based DL system has been developed (Li et al., 2020a), which applies optical character recognition techniques to extract data points in the VF and uses CNN models to modify the original ResNet18 to detect changes in the VF, resulting in the “iGlaucoma” mobile software. The DL system outperformed 6 ophthalmologists for different patterns in all three test sets and recognition pattern deviation probability map regions, showing its promise for clinical applicability.

Glaucoma diagnosis by VF measurement has become a common practice in clinical, and AI will have considerable potential in the field of automated VF measurement. However, the clinical generality of DL models from different studies, as well as the size of external test datasets and manual screening methods, affect the application of DL.

### 2.2 Fundus image

Artificial intelligence applied to fundus photography is a hot topic for researchers recently, because this methodology is able to perform targeted screening in different areas with simple devices, which makes the detection of early fundus lesions more convenient. Thus its mode of combining with AI for diagnosis has received attention from the initial detection of diabetic retinopathy (Gulshan et al., 2016; Abràmoff et al., 2018), and gradually applied to the diagnosis of glaucoma. AI focuses on problems including segmentation and detection of the optic nerve head, optic disc and RNFL to achieve accurate analyses of glaucoma fundus photographs. The early role of AI in glaucoma was comparatively simple, such as robust algorithms for quickly locating the optic disc region (Wang et al., 2017). This efficient kernelized least squares classifier extracts optic disc locations using vascular alignment, and its detection results on two digital retinal image datasets show that it has high Geometric Dilution Precision and speed. Then more DL systems and multi-integrated CNN models came out of the woodwork.

Among the numerous neural network structures for diagnosing glaucoma fundus photographs, the one more widely used is the ResNet structure. This DL model classifies fundus photographs with anatomical features of the upper and lower portions of the optic disc which are commonly used when diagnosing, with reduced training time. In 2018, (Christopher et al., 2018) used the transfer learning ResNet architecture to generate a DL algorithm that identifies glaucomatous optic neuropathy (GON). A subsequent study (Li et al., 2020b) using a DL model relying on ResNet101 demonstrated that identifying color fundus images with the neuroretinal rim region, RNFL defect areas (superior or inferior) and combining it with medical history information could better identify GON. However, several false negative results still affect the accuracy of the assay, e.g., pathologic or high myopia, age-related macular degeneration and diabetic retinopathy (Li et al., 2018b). The deep residual learning algorithm ResNet10 developed by Shibata et al. (2018) verified the accuracy of diagnosing glaucoma in high myopic eyes. It obtained an AUC value of 97.1% in the “G” (glaucoma) and “N” (normal eyes) groups and 96.4% in the “mG” (high myopia and glaucoma) and “mN” (high myopia and non-glaucoma) groups, which is markedly stronger than the residents (AUC 72.6%–91.2%). Recently, this network architecture has also been used in primary open-angle glaucoma (Fan et al., 2022). Experts took advantage of VF and optic disc information from 1,636 participants collected by the ocular hypertension treatment study over an average of 10 years to train the ResNet50 model and achieve high specificity on the test set beyond the study endpoint committee. To facilitate patient applications, DL algorithms have also been developed for smartphones using ResNet6 (Nakahara et al., 2022), which requires an accompanying D-Eye lens for fundus photo capture. Although it has some effectiveness in advanced glaucoma, its usage requires the flash to be continuously lit for 1 min against a dilated eye, which needs to be updated.

Several other neural network structures with different characteristics have also been employed for glaucoma detection. Deep convolutional neural algorithms in the Inception-v3 architecture identify optic nerve head features and GON from fundus images to facilitate early glaucoma referral diagnostic decisions (Phene et al., 2019). This algorithm was trained on individual pathological features and GON wholeness based on 86,618 fundus photographs and demonstrated AUCs between 0.855 and 0.945 on three test sets. Distinct from the traditional U-Net network, the SA module, namely, Scale-Attention Deep Learning Network, is inserted into the bridging connection to capture more scale features to interpret different structures and functions in retinal tissues (Hu et al., 2021). It can effectively segment 2D small sample retinal fundus images in order to determine glaucomatous fundus lesions. Another cycle generative adversarial network (CycleGAN) (Yoo et al., 2022) can connect information from the retro-ocular segment to the pre-ocular segment for detecting closed-angle glaucoma and has found that shallow anterior chamber depth is characterized by brighter areas around the optic disc and macula. Alternatively, one may choose to skillfully apply CycleGAN in combination with U-Net for retinal lesion localization (Zhang et al., 2022), where CycleGAN generates more available images and U-Net acts as a generator against the discriminator to generate the optimal solution, collaborating with the classifier to distinguish the domain of the input image. This demonstrates that a combination of different AI tools can improve diagnostic performance.

The ensemble model with its superior performance over the single model is now also highly preferred. Compared to a single model, the ensemble model combines the advantages of different AI image analyses as well as complements each other’s shortcomings. In 2020, (Ko et al., 2020) built an ensemble model TVGH-CNN merging a VGGNet-based CNN model and an SVM classifier to detect GON. For the easy-to-miss features of AI based on optic disc segmentation, such as increased vertical CorelDRAW and thinning of the upper and lower neuroretinal edges, it is possible to select a model via confidence scores, with SVM selected for low confidence in CNN, to achieve mid-to-late stage diagnosis of glaucoma. It achieved 95.0% accuracy and 94.2% specificity in the Drishti GS dataset. However, the classification accuracy is low and the generalizability is not high. But this method of assigning confidence scores provides a new way of thinking about the integration of models. Modeling several different neural networks to form an integrated system to automatically grade the severity of glaucoma is also a way forward for an ensemble system (Cho et al., 2021). Recently, a study has utilized a multimodal model to analyze the vertical cup-to-disc ratio and mean RNFL thickness to identify glaucoma in a myopic population (Lim et al., 2022). Where random forest, SVM, logistic regression, Ada-boost, k-nearest neighbors and a dense neural network were linked, and the images were categorized with the Xception model. This DL system was followed up with a web page for screening and telemedicine, covering a sizeable glaucoma suspect population.

Artificial intelligence scholars have focused on the convenience and trustworthiness of fundus photography, which can be implemented in less medically privileged areas, and have explored the possibilities of the combination of two technologies for accurate and efficient glaucoma diagnosis. This technology is now evolving from a single neural network to an ensemble model. Simple optic disc segmentation and vascular localization can determine whether disease is present or absent in GON detection and RNFL thickness measurement for grading glaucoma and identifying new fundus features.

### 2.3 OCT

Compared to fundus imaging, OCT has superior sensitivity and specificity. Recent innovations in OCT equipment have brought high-quality analytical data to the development of AI. Spectral domain OCT (SD-OCT) and scanning source OCT (SS-OCT) have improved axial resolution, enabling faster and more accurate acquisition of morphological features at the posterior end of the eye. Anterior segment OCT (AS-OCT) allows assessment of atrial angle opening and closing, anterior chamber depth as well as iris and lens to obtain biometric parameters (Ran et al., 2021). In contrast to traditional machine learning models, the DL model does not require segmentation and it can use the raw OCT data to classify and identify areas of lesion that are not readily detectable to humans.

**FIGURE 2:**
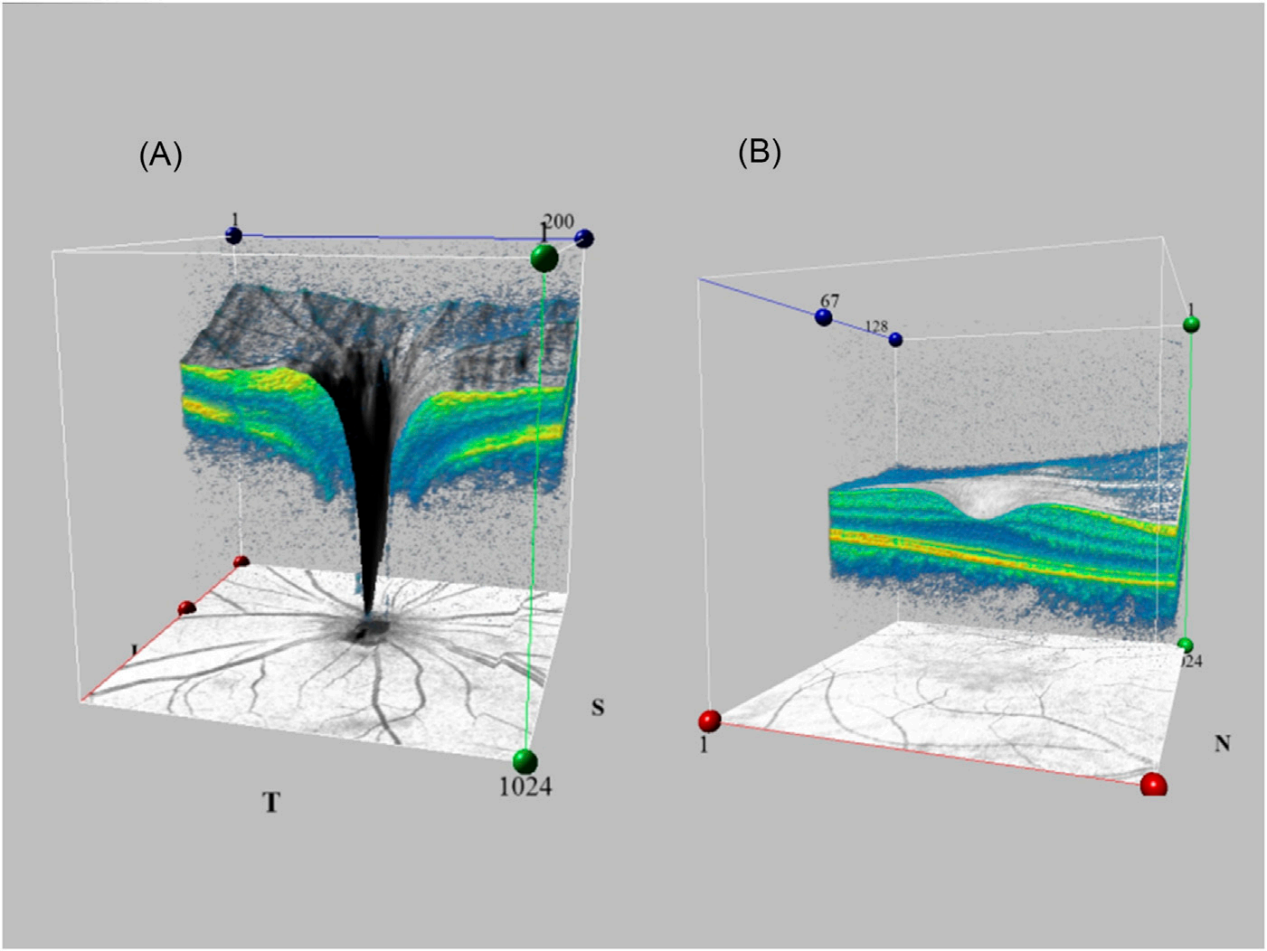
SD-OCT utilizes 3D capabilities for fundus scanning. Generating 3D fundus images by volume scanning and linear scanning of the feature site **(A)**. Optic disc region **(B)**. Macular region.

Nowadays SD-OCT is widely used in ophthalmology. A three-dimensional (3D) deep learning algorithm allows combination with SD-OCT to scan degeneration of the glaucomatous optic nerve head. Figure 2 shows the SD-OCT performing fundus scans using 3D capabilities. The first 3D deep learning system (Ran et al., 2019) which utilizes ResNet structures for glaucomatous lesion analyses in optic nerve head volume data collected by SD-OCT. Compared to 2D models, it uses 3D deep learning algorithms to obtain better results. Its diagnosis relies on accurate segmentation of the retinal layers, as well as quantification of the RNFL and macular ganglion cell complex. Later scholars (Noury et al., 2022) attempted to formulate a 3D convolutional neural network and trained it to discriminate glaucoma on datasets from Stanford University, Hong Kong, India, and Nepalese sources, responding to its good judgment in OCT images of different ethnic groups. For glaucoma diagnosis, the lamina cribrosa region is highlighted. This is consistent with clinical parameters like cup diameter/volume or rim area/volume. Other scholars wanted to develop a multi-task 3D deep learning model to detect GON and other fundus lesions like myopia in relation to glaucoma diagnosis (Ran et al., 2022). ResNet architecture was developed with multi-input CNNs and multi-channel variational autoencoders, which were applied for internal and external validation, with results outperforming RNFL thickness. CNNs have been trained before by using the macular RNFL thickness and ganglion cell complex layer thickness on images (Asaoka et al., 2019), which performed better than random forest and SVM. Rather than being limited by errors in manual classification of subjective markers, it saves even more manpower and time (Medeiros et al., 2021). Subsequently, researchers have attempted to develop a model for predicting the development of glaucoma by combining fundus photographs with OCT in a “machine-to-machine” model (Medeiros et al., 2019). This model continues to use the ResNet34 architecture to consecutively predict the mean RNFL thickness, achieving a mean absolute error (MAE) of 7.39 μm. RNFL thickness values for all sectors “CNNA” combined with temporal sectors “CNNT” using SD-OCT circle scans can also be used to measure the central 10° visual field map in glaucoma patients (Kamalipour et al., 2023). Or AI can use the additional information collected by SD-OCT to form an overall DL model to evaluate visual function, such as ganglion cell layer, ganglion cell-inner plexiform layer, inner plexiform layer and macular ganglion cell layer (Christopher et al., 2021). Lee et al. (Lee et al., 2020a) developed a hybrid deep learning model algorithm that utilized Inception-ResNet-v2 to extract features and paired it with SVM for regression and classification problems. The results with red-free RNFL photographs measurement macular ganglion cell-inner plexiform layer thickness showed a correlation coefficient of *r* = 0.739 and a consistency metric of MAE = 4.76 µm with the true measurements. This suggests that hybrid DL models also hold good promise for applications in OCT.

**FIGURE 3:**
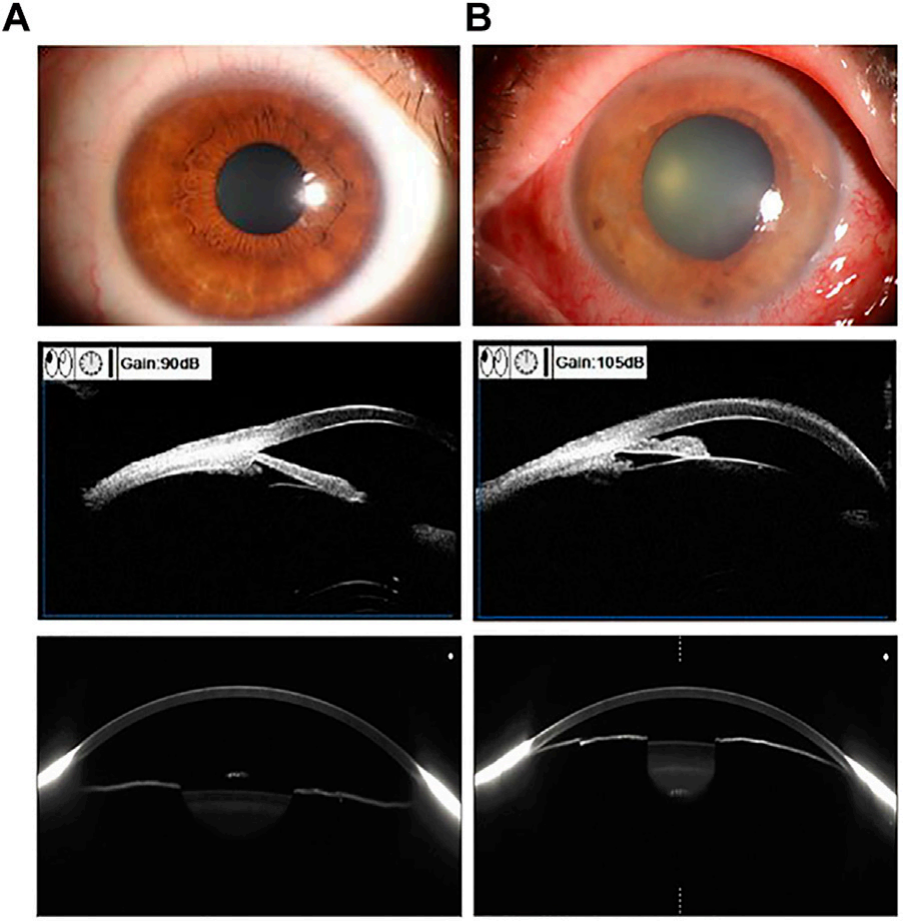
Using Anterior segment OCT (AS-OCT) in comparison with other imaging of the anterior segment of the eye. Examples of the preocular segment using ocular surface photography (first row), ultrasound biomicroscopy (second row), and AS-OCT scan (third row). Vertical column **(A)**. Normal eye **(B)**. Angle-closure eye.

Traditional detection of the preocular segment using AS-OCT depends on the ophthalmologist’s refinement of the image and identification of the scleral spur. Artificial intelligence can automatically perform the extraction of features from AS-OCT such as thick peripheral iris roll, plateau iris and expanded lens vault in the anterior segment and reduce the errors caused by manual recognition through feature extraction. This AI system can detect anterior chamber angles more accurately than other auto-angle closure detection systems. Figure 3 shows the comparison of AS-OCT with other imaging modalities of the anterior segment. By placing long short-term memory neural networks, which captures temporal information in images, in the last pooling layer of a trained ResNet model (Hao et al., 2022), a DL model was constructed to process image data and investigate the relationship between dynamic iris changes and primary angle-closure glaucoma. Xu et al. (Xu et al., 2019) formulated three competing multiclass CNNs to compute binary probabilities of atrial angle closure (Shaffer class 0 or 1). In the end the ResNet18 classifier obtained superior performance. It helps clinical judgment of angle opening and closing by using accurate calculation power, which saves time and improves accuracy. The DL model also allows for the development of an automated digital gonioscopy system to simulate static and dynamic goniometry at a level not inferior to that of a clinician (Li et al., 2022b). Alternatively, a sliding window-based regression task for locating the anterior atrial angle can be created to automatically perform angle detection using three parallel sub-networks to process data and extract image features (e.g., anterior segmental allostructure, iris structure, atrial angle structure) from the AS-OCT output. The Chinese American Eye Study has utilized this theory to develop a DL model for detecting the atrial angle that has been tested in communities of different ethnicities (Randhawa et al., 2021). AS-OCT combined with AI can also analyze features that are not noticed by humans to facilitate the adoption of unsupervised learning. For example, setting the latent space size of the *β*-variational autoencoder to 6 and the beta value to 53 in the study by Shon et al. (2022a); Shon et al., 2022b) enables the extraction of low-dimensional latent variables, which can then be converted into shallow and understandable features.

As the machines are updated, new features are being created to fill the gaps in the development of modern OCT, which can be combined with a variety of ophthalmic examination instruments to detect multiple diseases simultaneously and even explore the pathogenesis of glaucoma in the microscopic cellular world. For example, an hybrid deep learning model can be linked with a single wide-field OCT for quadrant analysis to differentiate glaucoma (Muhammad et al., 2017), the custom DL architecture LightOCT can classify various diseases of the eye in public datasets (Butola et al., 2020), and SS-OCT is combined with a DL model to detect peripheral anterior adhesions (Yang et al., 2021). Zadeh et al. (Soltanian-Zadeh et al., 2021) used adaptive optical AO-OCT with weakly supervised deep learning WeakGCSeg to automatically segment cells in order to study the cellular level characteristics of retinal ganglion cells, using click-points weak supervision to generate a fast, high-throughput detection system.

From the perspective of public health, OCT belongs to a category of expensive testing equipment, which is not as widely tested everywhere on a large scale as fundus imaging. But because of its precision, it is ideally suited to provide AI with more specific ocular features, and because of the addition of AI, it also makes OCT technology more labor efficient. They complement each other. In the future, it is likely that SD-OCT with its universality and AS-OCT with its immediacy will have a large market share in the field of glaucoma research. But other OCT techniques will receive more attention and exploration on the strength of their unique ability to retrieve information. Table 1 shows the deep learning studies for glaucoma diagnosis using OCT images as input.

**TABLE 1:** Summary table of deep learning studies for glaucoma diagnosis with OCT images as input.

References	Year	Model	Dataset			Aim	Result
Ran et al.	2019	ResNet	GON/No GON			detect GON	Primary validation: AUROC: 0·969, sensitivity: 89%, specificity: 96%, accuracy: 91%
	—	—	Training, testing, and primary validation dataset	2926/1951		—	External validation: AUROC: 0·893–0·897, sensitivities: 78%–90%, specificities: 79%–86%, accuracies: 80%–86%
	—	—	External validation dataset	1434/610		—	—
Noury et al.	2022	DiagFind	Glaucoma/Non-glaucoma			manifest glaucoma	AUC: perimetric glaucoma
							Stanford: 0.91
							Hong Kong: 0.80
							India: 0.94
							Nepal: 0.87
	—	—	Training	1022/542		—	—
	—	—	Validation	142/61		—	—
	—	—	Test	453/241		—	—
	—	—	External validation dataset	1642/1035		—	—
Ran et al.	2022	ResNet	yes GON and yes MF/no GON and yes MF/yes GON and no MF/no GON and no MF			GON	AUROC
						MF	GON: Internal validation 0.949
							External testing dataset 0.890–0.950
	—	—	Training	1679/890/629/721		—	MF: 0.855–0.896
	—	—	Tuning	195/163/32/70		—	—
	—	—	Internal validation	205/114/36/99		—	—
	—	—	External testing dataset	1347/515/677/777		—	—
Asaoka et al.	2019	Deep learning	Glaucoma/Non-glaucoma			early glaucoma	AUC
							Pretraining: 93.7%
							Without pretraining: 76.6%–78.8%
			Pretraining	1371/193		—	—
			Training	94/84		—	—
			Test	114/82		—	—
Medeiros et al.	2021	ResNet50	86 123			progressive glaucomatous changes over time	AUC: 0.86
Medeiros et al.							
	2019	ResNet34	Normal/Suspect/Glaucoma			quantify glaucomatous structural damage	MAE: 7.39 μm
							AUC: predictions: 0.944
							actual measurements: 0.940
	—	—	Training	3982/13 410/9136		—	—
	—	—	Test	877/3345/2070		—	—
Kamalipour et al.	2023	CNN_A_	Normal/Suspect/Glaucoma			estimate central 10° visual field	MAE
							CNN_A_: 4.04 dB
	—	CNN_T_	Training and Validation	174/367/623		—	—
	—	LR	Test	20/71/110		—	—
Christopher et al.	2021	ResNet50	10-2 Visual Field/24-2 Visual Field			estimating visual function	10-2
							R^2^ MD:0.82
							PSD: 0.69
							MAE MD: 1.9 dB
	—	—	Training	2131/277		—	24-2
							R^2^ MD:0.79
							PSD: 0.68
							MAE MD: 2.1 dB
	—	—	Test	2674/325		—	—
Lee et al.	2020a	HDLM	Normal/Suspect/Glaucoma			predicts macular ganglion cell-inner plexiform layer thickness	MAE: 4.76 μm
	—	—	292/109/388			—	—
Hao et al.	2022	ResNet + LSTM	Glaucoma/Non-glaucoma			angle-closure screening	AUC
							Casia dataset: Images 0.766; Original videos 0.820; Aligned videos 0.905.
	—	—	159/210			—	Zeiss dataset: Images 0.767; Original videos 0.837; Aligned videos 0.919
Xu et al.	2019	ResNet18	Open angle/Closed angle			detect gonioscopic angle closure and primary angle closure disease	AUC: gonioscopic angle: 0.928
							disease: 0.952
	—	—	Cross-validation	1632/1764		—	—
	—	—	Test	311/329		—	—
Li et al.	2022b	ResNet34	Task I/Task II			Task I (1) narrow iridocorneal angles	Task I
							AUC: 0.943, sensitivity: 0.867, and specificity: 0.878
	—	—	Training	4515/378		Task II (2) peripheral anterior synechiae	Task II
							AUC: 0.902, sensitivity: 0.900, and specificity: 0.890
	—	—	Internal validation	1101/376		—	—
	—	—	External testing	2222/102		—	—
Randhawa et al.	2021	ResNet18	Open angle/Closed angle			detect gonioscopic angle closure	AUC: 0.894–0.922
	—	—	CHES train	1764/1632		—	—
	—	—	CHES test	329/311		—	—
	—	—	Singapore	570/9595		—	—
	—	—	USC	66/234		—	—
Shon et al.	2022a	β-VAE	Training	1692		extract a low-dimensional latent structure	mean values of visual field index: 86.4%
							mean deviation: −5.33 dB
	—	—	Validation	419		—	—
Shon et al.	2022b	VAE	Training	1692		Analysis the latent structure	Among the symmetrical latent variables, the first three and the last demonstrated easily recognized features.
	—	—	Validation	419		—	—
Muhammad et al.	2017	HDLM	Glaucoma/Health or suspects			Distinguish glaucoma eyes	accuracy: 63.7%–93.1%
	—	—	57 eye/45 eye			—	—
Butola et al.	2020	LightOCT	Choroidal neovascularization/Diabetic macular edema/Drusen/Normal			Distinguish glaucoma eyes	accuracy: 96%
	—	—	Training	27 206/11 349/8617/51 140		—	—
	—	—	Test	250/250/250/250		—	—
Yang et al.	2021	InceptionResNetV2	Open angle/Closed angle			detect the static gonioscopic angle closure and peripheral anterior synechia	static gonioscopic angle closure
							AUC: 0.963 sensitivity: 0.929
							specificity: 0.877
	—	—	Training		3 4705/1 5945	—	appositional from synechial angle closure
							AUC: 0.873
							Sensitivity: 0.846
							Specificity 0.764
	—	—	Validation		8037/3254	—	—
	—	—	Test		7860/3024	—	—
Soltanian-Zadeh et al.	2021	WeakGCSeg	Training samples/Testing samples			Cell-level quantitative features of retinal ganglion cells	WeakGCSeg is on par with or superior to human experts and is superior to other state-of-the-art networks.
	—	—	Subject 1 (IU/IU)		Healthy: 7:14/1:2	—	—
	—	—	Subject 2 (IU/IU)		Healthy: 7:14/1:1	—	—
	—	—	Subject 3 (FDA/FDA)		Healthy: 3:4-5/1:1-2	—	—
					Glaucoma: 4:8/1:2		
	—	—	Subject 4 (IU/FDA		Healthy: 8:16/4:6	—	—
			FDA/IU		Healthy: 4:6/8:16		
			IU + FDA/IU + FDA)		Healthy: 9:16–17/9:16–17		

● ResNet residual network, GON, glaucomatous optic neuropathy; AUROC, area under the receiver operating characteristic; AUC, area under curve; MF, myopic features; MAE mean absolute error; CNN, convolutional neural network; LR, ordinary least squares linear regression models; MD, mean deviation; PSD, pattern standard deviation; HDLM, hybrid deep learning method; LSTM, long short-term memory; CHES, the Chinese American Eye Study; USC, the University of Southern California; VAE variational auto-encoder; IU, the Indiana University; FDA, the U.S., food and drug administration.

## 3 Prediction models in glaucoma

Keeping track of a patient’s progress can be of great significance in preventing blindness and delaying the condition. Glaucoma requires close monitoring of longitudinal case information by doctors and timely medical intervention to salvage the nerve and VF damage caused by high intraocular pressure. Although prediction and diagnosis are similar by virtue of the detection tools, the conception of the DL model extraction, analysis and output content is not identical. They target different sites and severity of lesions in different periods of glaucoma, and the methods of analyses used are not uniform. It is widely known that glaucoma is closely related to VF, so the prediction of glaucoma in all aspects is largely focused on the prediction of VF as well. However, it has undergone some processes to be perfected. Some researchers (Eslami et al., 2023) investigated the accuracy of the previously emerged CNN model and recurrent neural network model in predicting VF changes over time. Although exerting some power, both models showed errors in predicting patients with severe glaucoma, like grossly underestimating the degree of deterioration in VFs loss and working poor in patients with large changes in VFs at baseline and follow-up. These may affect the clinical applicability. The generalized Variational Autoencoder DL model (Berchuck et al., 2019) ameliorates these problems to some extent. It uses a lower dimensional latent space representation of a higher dimensional VF image to output a resultant prediction via an arbitrary non-linear mapping. It was learned and tested on 29,161 VFs with good results.

In subsequent studies, the researchers predicted the course of glaucoma by using various auxiliary tests in the AI analyses to predict VF changes. In previous years, scholars first used fundus photographs to predict the progression of glaucoma (Thakur et al., 2020). They found the AUC predicted from fundus photographs 4-7 years before onset was 0.77, 1-3 years predicted 0.88, and 0.95 for post-onset diagnosis. The closer to the time of onset the more obvious the lesion is the easier it is to detect by AI. Additional studies (Lee et al., 2020b) have transformed qualitative structural data (optic disc photograph) into quantitative functional data (standard automated perimetry, mean deviation), predicting standard automated VF measurements from single-field optic disc photographs. A neural architecture search network (NASNet) was used to extract fundus features and predict VF progression. There are other studies that have used machine-to-machine approaches, such as (Lee et al., 2021) who trained deep learning algorithms in OCT images to predict longitudinal changes in RNFL thickness on fundus images to explore whether it could predict the future development of glaucomatous VFs. A longitudinal survival model was used for this retrospective cohort study, controlling for other confounding factors (e.g., age, mean intraocular pressure, etc.), and the DL system still accurately predicted regression in glaucoma. The ResNet6 architecture allows the exploitation of a multiple linear regression model that, after pre-training on a larger dataset followed by fine-tuning and transfer learning, can also perform well on a smaller training set, achieving a smaller mean absolute error, which is advantageous when applied to VF prediction for fundus imaging. These technologies will also gradually come into our lives, with a glaucoma prediction system for smartphones already in development (Li et al., 2022a).

The application of OCT images to predict glaucoma also focuses on the evaluation of the VFs. Predicting changes in the HFA 10-2 visual field based on macular retinal layer thickness measured by SD-OCT, and HFA 24-2 test values, both ResNet and VGG algorithms can be applied (Christopher et al., 2020; Asano et al., 2021). It is also possible to analyze RNFL, ganglion cell layer and inner plexiform layer, outer segment and retinal pigment epithelium using the unsupervised learning pattern-based regularization method to determine the 10° central field of view (Hashimoto et al., 2021). Or use tensor regression (CNN-TR) (Xu et al., 2020a) to develop a model with higher-order multiple regression to reduce the number of parameters and improve accuracy. It replaces fully connected neural networks and vectorisation operations and has the relative advantage of predicting the central 10° field of view. In addition to SD-OCT, SS-OCT can also be employed. SS-OCT (Park et al., 2020) combined with the Inception architecture allows for VF prediction, but with decreasing accuracy as glaucoma progresses. Supplementary Table S1 summarizes the deep learning studies used for glaucoma prediction.

At the current stage, prediction of structures and functions is not yet complete and multimodal detection tools are still being bred. To get a better trend, it is necessary to combine structure and function, collect more clinically relevant data from patients and incorporate multiple perspectives to analyze the outlook for disease progression. For future clinical applications, it may be more promising to investigate early prediction before the onset of the disease.

## 4 Limitations and further advancements

### 4.1 Limitations

With the introduction of artificially intelligent diagnostic and predictive models, our review opens a door to a new approach to the detection of glaucoma. The logic behind studying these technologies is that they allow for direct clinical diagnosis, the timely identification of patients requiring referral or surgical intervention is critical for glaucoma specialists, and AI can assist with such steps to reduce the loss of vision and VFs in patients. Today’s epidemic of SARS-CoV-2 and the crowding of medical resources are challenging the healthcare system, requiring AI to combine with medicine to facilitate each other’s development and provide more sophisticated technology for increased medical needs. Visualization of the AI analysis reveals that its segmentation and judgments are often in most of the same locations as those used by ophthalmologists in their diagnoses, but also in some of the features that have not been identified by humans. This will be a key component of future developments, which will better reveal the pathological mechanisms of disease and obtain more information from the raw images. Yet the growth of AI in recent years has also revealed a number of problems.

1) Standard dataset creation It is about whether AI can be developed or even have practical effects. Firstly, the dataset is homogeneous in terms of features. Because the datasets being used are publicly available (Camara et al., 2022), the classification of images lacks specificity that is often consistent with homogeneous diseases or populations, making it difficult to ensure the fairness of the validated results. Secondly, the utilization of clinical data is very low. Although there have been some studies that have utilized clinical features of disease with some personalization, AI still has low usage of clinical records, resulting in little information for grading prognosis of diseases. Third, the quality of images varies. Public datasets are often created for large-scale disease screening or treatment evaluation, and their images may be all-encompassing but lack classification and labeling. The quality of photography also varies, with no uniform standards to regulate it. Private datasets tended to be difficult to access and had insufficient images (<10,000). Finally, the AI developed by different datasets lacks comparability. DL models trained and tested with large datasets are not necessarily more accurate than with small datasets (Zheng et al., 2019). Moreover, transfer learning without adjustment is likely to fail in the real world, and the AUC is not as reliable and will be higher in smaller datasets.
2) Standardized norms and guidelines Any medical instrument must undergo multiple rounds of experimental validation and production of manuals for reference before it can be clinically applied. So does AI. However, we only hear the call of the market, but do not see the preparation for it. Although some countries have already proposed specifications (Bossuyt et al., 2003), an international consensus standard is needed to face the rapidly evolving AI systems. The definition of criteria for accuracy and specificity, the uniformity of testing, the fixed population to which it can be applied, and the long-term follow-up system make up a complete evaluation system.
3) Single and simple usage Glaucoma is a complex neurodegenerative disease that requires both structural and functional evidence for a definitive diagnosis. The numerous and lengthy tests are burdens for both doctors and patients, and AI has not yet evolved to a point where a definitive diagnosis can be made with simple one method; it can only improve on one of the existing tests to a certain extent, which is the direction to be considered in the future.
4) Multi-ethnic applications Although technology has no borders, people of different nationalities have different physiques. How the invented AI can serve more people to expand its popularity, if it has good sensitivity and specificity in different people, requires more cross-border, multi-center experimental studies.
5) Black Box Theory In clinical work, it is difficult to gain patients’ trust if they cannot understand the treatment tools, and only conclusions can be drawn about the new disease features explored by CNNs, without complete clarity of the inference process. The “black box” nature of AI makes it uninterpretable, and even with the application of feature visualization techniques such as Class Activation Mapping, the completed analysis process of a CNN is still not available. This is the area that needs to continue exploration.

### 4.2 Further advancements

Anything that develops faces more than one type of problem, but this is exactly the prospect necessary for growth. There are already some scientific answers to the questions we discussed, for example, for dataset updates, articles have been reported using Generative Adversarial Networks to modify synthetic real images to enrich existing datasets without compromising patient privacy. Regarding image quality it has also been reported that CycleGAN’s tool can eliminate artifact interference. The existing research in AI shows that there is a great interest in new disease features perceived by DL models, which may be an important clue to investigate the source of the disease and deserves to be studied in depth. In response to the issues raised, dataset optimization, including the establishment of multicenter, large sample, and high validity datasets is the determined growth prospect. Simplification of the existing diagnostic methods in a single way, so that the created AI system serves more diverse people is also a development direction to be worked on. Uniform rules and guidelines for assisted development require global consultation and exchange of ideas. Apart from the aforementioned, we believe that the development of telemedicine and virtual reality technology will become a hot prospect for ophthalmology and even the whole medical field under the conditions of today’s social communication networks and electronic equipment hardware. There has been some progress in the research of AI and telemedicine (Nikolaidou and Tsaousis, 2021), and the integration with virtual reality technology (Ma et al., 2022) is still on the rise. In addition, glaucoma is a genetically linked genetic disorder and should be subject to early genetic screening and referral for further precise diagnosis and intervention. Genetic diagnosis combined with AI technology development is another way to obtain critical evidence that improves diagnostic efficiency and allows for better patient prognosis. These fields of research will certainly bring the technology of AI for glaucoma diagnosis and prediction to maturity and canonicalization.

## 5 Conclusion

Artificial intelligence is evolving rapidly in the field of ophthalmology, and since previous reviews on glaucoma with AI are not perfect, we have written this targeted review to explore the development of AI models in the field of glaucoma diagnosis and prediction in recent years, especially the revolution of DL models. We present the current state of research and found that scholars have mainly used HFA, fundus photography and OCT, which are commonly used in ophthalmology to collect image data. Using network structures such as ResNet, VGG, U-Net, Inception, CycleGAN and ensemble convolutional neural that can explore deeply to extract image features. Dataset quality, uniform rules and guidelines, single and simple usage, multi-ethnic applications and black box effect will be the critical issues to be addressed. Issues such as genetic screening for glaucoma and telemedicine could be promising opportunities.
