# Supplementary material for: The application of artificial intelligence in glaucoma diagnosis and prediction
Source: Front Cell Dev Biol. 2023 May 4;11:1173094. doi: 10.3389/fcell.2023.1173094 (PMC10192631; doi:10.3389/fcell.2023.1173094)
Supplement: Supplementary file 1 [file Table1.DOCX]

**Supplementary Table 1.** Summary table of deep learning studies for glaucoma prediction.

| **Reference** | **Year** | **Model** | **Input** | **Dataset** | | **Prediction** | **AUC** | **MAE** | **RMSE** |
| --- | --- | --- | --- | --- | --- | --- | --- | --- | --- |
| Eslami et al. | 2022 | CNN(CascadeNet-5); | HFA 24-2 VFs | Glaucoma: CNN/RNN | | Pointwise VFs |  | PMAE: Mwen(CNN): 2.21-2.24 dB |  |
|  |  | RNN |  | Training | 43 498/19 544 |  |  | PMAE: Mpark(RNN): 2.56-2.61 dB |  |
|  |  |  |  | Validation | 4350/1954 |  |  |  |  |
|  |  |  |  | Test | 10 875/4886 |  |  |  |  |
| Berchuck et al. | 2019 | VAE | SAP 24-2 VFs | Non-glaucoma/Suspect/Glaucoma | | Rates of VFs progression; |  | 5.14 dB |  |
|  |  |  |  | Training | 547/58/62 | Future VFs |  |  |  |
|  |  |  |  | Validation | 1793/222/206 |  |  |  |  |
|  |  |  |  | Test | 768/83/93 |  |  |  |  |
| Thakur et al. | 2019 | MobileNetV2 | CFPs | Non-glaucoma/4-7 years prior onset/1-3 years prior onset/After onset | | Glaucomatous VFs development | 4-7 years prior to disease onset: 0.77; |  |  |
|  |  |  |  | Training | 28 082/816/886/2775 |  | 1-3 years prior to disease onset: 0.88 |  |  |
|  |  |  |  | Validation | 6195/180/195/612 |  | after onset: 0.95 |  |  |
|  |  |  |  | Test | 7021/204/222/694 |  |  |  |  |
| Lee et al. | 2020 | NASNet | ODPs | Non-glaucoma/Glaucoma | | MD of SAP | 0.953 | 1.94 dB |  |
|  |  |  |  | Training+Validation | 483/517 |  |  |  |  |
|  |  |  |  | Test | 81/119 |  |  |  |  |
| Lee et al. | 2021 | M2M | CFPs | Glaucoma | 1072 | Glaucomatous VFs development |  |  |  |
| Li et al. | 2022 | DiagnoseNet(U-Net; EfficientNet-B0); | CFPs | Glaucoma diagnosis/Glaucoma onset prediction/Glaucoma progression prediction | | Glaucoma incidence and progression | External test set 1/External test set 2 |  |  |

**Supplementary Table 1. Continued**

| **Reference** | **Year** | **Model** | **Input** | **Dataset** |  | **Prediction** | | | **AUC** | | **MAE** | | **RMSE** |  |  |
| --- | --- | --- | --- | --- | --- | --- | --- | --- | --- | --- | --- | --- | --- | --- | --- |
|  |  | PredictNet(U-Net;ConvNet) |  | Training | 20 872/10 357/3003 |  | | | Glaucoma diagnosis 0.94/0.91; | |  | |  |  |  |
|  |  |  |  | Validation | 3182/1191/422 |  | | | Glaucoma incidence prediction 0.89/0.88; | |  | |  |  |  |
|  |  |  |  | External test set 1 | 6162/955/337 |  | | | Glaucoma progression prediction 0.87/0.88 | |  | |  |  |  |
|  |  |  |  | External test set 2 | 824/719/513 |  | | |  | |  | |  |  |  |
| Christopher et al. | 2020 | ResNet50 | SD-OCT images | GVFD+/GVFD- | | Severity of GFVD | | | 0.88 | | MD:2.5dB | |  |  |  |
|  |  |  |  | Training | 919/704 |  | | |  | |  | |  |  |  |
|  |  |  |  | Validation | 54/41 |  | | |  | |  | |  |  |  |
|  |  |  |  | Test | 108/83 |  | | |  | |  | |  |  |  |
| Asano et al. | 2021 | VGG19; | SD-OCT images； | Non-glaucoma/Glaucoma | | Central 10 degrees VFs | | |  | | ResNet-adjust: 5.3 dB; | |  |  |  |
|  |  | ResNet152 | TD values of the HFA 10-2 VF test points | Training | 90/558 |  | | |  | | VGG-adjust: 5.4 dB | |  |  |  |
|  |  |  |  | Testing | n/105 |  | | |  | |  | |  |  |  |
| Hashimoto et al. | 2022 | ResNet; | SD-OCT images | Non-glaucoma/Glaucoma | | Central 10 degrees VFs | | |  | |  | | ResNet: 2.84 dB; |  |  |
|  |  | SVM; |  | Training | 86/505 |  | | |  | |  | | SVM: 5.65 dB; |  |  |
|  |  | MLR |  | Test | n/160 |  | | |  | |  | | MLR: 6.96 dB |  |  |
| Xu et al. | 2020 | CNN-PR(VGG16); | SD-OCT images | Non-glaucoma/Glaucoma | | Central 10 degrees VFs | | |  | |  | | CNN-TR: 6.32 dB; |  |  |
|  |  | CNN-TR(VGG16); |  | fivefold cross-validation | 86/505 | | |  | |  | |  | CNN-PR: 6.76 dB; | | |
|  |  | SVR; |  |  |  | |  | |  | |  | | SVR: 7.18 dB; | |  |
|  |  | MLR |  |  |  |  | | |  | |  | | MLR: 8.56 dB |  |  |

**Supplementary Table 1. Continued**

| **Reference** | **Year** | **Model** | **Input** | **Dataset** |  | **Prediction** | **AUC** | **MAE** | **RMSE** |
| --- | --- | --- | --- | --- | --- | --- | --- | --- | --- |
| Park et al. | 2020 | Inception-ResNet-v2; | SS-OCT images | Non-glaucoma/Early glaucoma /Advanced glaucoma | | Future VFs |  |  | Inception-ResNet-v2： 4.44 dB; |
|  |  | Inception-v3; |  | Training | Unlabeled 1998 |  |  |  | Inception-v3: 4.78 dB; |
|  |  | Inception-v4 |  | Validation | Unlabeled 222 |  |  |  | Inception-v4: 4.85 dB |
|  |  |  |  | Test | 105/109/91 |  |  |  |  |

Al artificial intelligence, CNN convolutional neural network, RNN recurrent neural network, VAE variational auto-encoder, M2M machine to machine, NASNet neural architecture search network, ResNet residual network, VGG visual geometry group, SVM support vector machine, MLR multiple linear regression, SVR support vector regression, HFA Humphrey Field Analyzer, VF visual field, SAP standard automated perimetry, CFP color fundus photograph, ODP optic disc photograph, SD-OCT spectral-domain optical coherence tomography, TD total deviation, SS-OCT swept-source optical coherence tomography, GVFD glaucomatous visual field damage, MD mean deviation, RNFL retinal nerve fiber layer, AUC area under curve, MAE mean absolute error, RMSE root mean squared error, PMAE pointwise mean absolute error.
